# Editorial: Multifaceted Genes in Amyotrophic Lateral Sclerosis-Frontotemporal Dementia

**DOI:** 10.3389/fnins.2021.680185

**Published:** 2021-04-23

**Authors:** Francesca Luisa Conforti, Alan Edward Renton, Henry Houlden

**Affiliations:** ^1^Department of Pharmacy, Health and Nutritional Sciences, University of Calabria, Rende, Italy; ^2^Ronald M. Loeb Center for Alzheimer L Disease, Icahn School of Medicine at Mount Sinai, New York, NY, United States; ^3^Department of Neuromuscular Disorders, Institute of Neurology, University College London, London, United Kingdom

**Keywords:** amyotrophic lateral sclerosis, ALS-FTD spectrum, genetics, genomics, omics

Amyotrophic lateral sclerosis-frontotemporal dementia (ALS-FTD) is a heterogeneous, multi-factorial, and multi-system disease spectrum currently lacking effective drug treatments. The fields of ALS-FTD genetics and genomics have greatly expanded since the first disease gene *SOD1* was identified in 1993 (Rosen et al., 1993). The advent of high-throughput next generation sequencing technologies has enabled systematic genome-wide interrogation of genetic variation, implicating disease-causing and disease-modifying genetic loci and improving our understanding of the diverse pathogenic basis of ALS-FTD. Over 30 genetic loci have been reproducibly linked or associated with ALS-FTD and novel loci continue to be identified (Chia et al., 2018; Guerreiro et al., 2020). It is now recognized that ALS and FTD constitute a disease spectrum or syndrome rather than distinct disorders. This scenario exemplifies the emerging observation of phenotypic pleiotropy, where mutations in the same gene give rise to diverse phenotypes, further increasing the complexity of genotype-phenotype correlation.

In 2011, the discovery that the *C9orf72* GGGGCC repeat expansion (C9-RE) is the most frequent genetic cause of ALS and FTD definitively consolidated the hypothesis that the two diseases belong to the same clinicopathological spectrum (DeJesus-Hernandez et al., 2011; Renton et al., 2011). Repeat expansions have emerged in recent years as major contributors to motor neuron degeneration and with the advent of long-read sequencing, further expansions are likely to be discovered. Intermediate-length CAG repeat expansions in both *ATXN1* (Conforti et al., 2012; Tazelaar et al., 2020) and *ATXN2* (Elden et al., 2010) have also been associated with an increased risk of developing ALS. Mutations in *OPTN, VCP, SQTM1, MATR3*, and *NEK1* have offered insight into the connections between ALS-FTD and seemingly unrelated clinical disorders such as Paget's disease and myopathy (Chia et al., 2018). Recently, *KIF5A*, a gene previously linked to two rare neurodegenerative disorders, hereditary spastic paraplegia type 10 and Charcot-Marie-Tooth type 2, has been definitively linked to ALS (Brenner et al., 2018; Nicolas et al., 2018). Taken together, these and other genes have highlighted the complex genetic architecture of ALS-FTD, with many genes in seemingly unrelated or distantly related physiological pathways producing a similar phenotype.

This Research Topic includes significant focus on the C9-RE in ALS and FTD patients. Trojsi et al. studied the C9-RE in a large Italian ALS cohort. They reported C9-RE carriers exhibit ALS symptoms clinically distinct from sporadic ALS (sALS) patients and, found male but not female expansion carriers have decreased survival, suggesting a potential link between sex and disease progression. Esselin et al. described a large French ALS-FTD cohort with C9-RE. They observed C9 patients have an earlier age of onset compared to sALS patients, familial index cases and their siblings have an earlier age of onset compared to their parental generation suggesting anticipation, a predominant female transmission, and a high frequency of suicides in relatives. Trageser et al. reviewed the role of immune cell activation in ALS-FTD in the context of the C9-RE, providing an overview of C9-linked ALS-FTD pathogenesis and the interplay of these cellular events with the immune system. The authors suggested the C9-RE mediates neuroinflammatory mechanisms that significantly contribute to pathogenesis and represent promising new therapeutic approaches.

This Topic also concentrates on overlapping and discordant genetics across ALS, FTD and other disorders. Tripolszki et al. contributed with their first comprehensive genetic analysis of the Hungarian ALS population, highlighting the necessity for large-scale studies to distinguish true causative genetic variants from irrelevant ones and accurately uncover the genetic pattern of ALS. Abramzon et al. described genes involved both in ALS and FTD as key players in dysfunctional pathways such as RNA processing, autophagy, vesicle trafficking, mitochondria, and protein homeostasis. Due to such significant genetic overlap between ALS and FTD, the authors recommended looking in FTD cases for mutations in ALS genes and vice-versa. On the other hand, Ranganathan et al. highlighted that some genes are linked with only ALS or FTD, such as *SOD1* and *MAPT*. This distinction is reflected in the neuropathology, because most types of monogenic ALS, C9-FTD, and GRN-FTD are characterized by TDP-43-positive inclusions but *SOD1*-ALS and *MAPT*-FTD are not, underscoring the need to consider disease subtypes when conducting biomarker and therapeutic research. The authors discussed using next generation sequencing to identify multiple variants in disease-associated genes within an individual, emphasizing the importance of genomic data to facilitate a precision medicine approach for treating ALS-FTD. Furthermore, Broce et al. discussed how shifting our focus from studying ALS and FTD in isolation to identifying the common and distinct biological mechanisms that drive these diseases will improve treatment discovery and therapeutic development. Hence whole genome sequencing of large international ALS-FTD cohorts will begin to fully understand the genetic contribution to disease, particularly when large collaborative cohorts are sequenced such as in project MinE. Rich et al. suggested genome-wide association studies and rare variant association studies represent an attractive option for novel gene discovery because they do not require prior knowledge or hypotheses. Lower-penetrance alleles identified via association studies may inform important components of future combinatorial gene-targeted therapies.

Additionally, the field shows increasing interest in omics bioinformatic analysis to elucidate ALS complex molecular architecture and its role in clinical heterogeneity. Lin et al. compared gene expression profiles of sALS and control motor neurons to discover differentially expressed genes then identified pathways and regulators underlying sALS. They found differentially expressed genes are enriched for the extracellular matrix and implicated the NF-κB regulatory pathway in sALS pathogenesis. Finally, Morello et al. discussed the most significant contributions of omics approaches (genomics, transcriptomics, proteomics, and metabolomics) in unraveling the biological complexity of ALS, highlighting how holistic systems biology approaches and multi-omics data integration are ideal to provide comprehensive characterization of patient-specific molecular signatures that could potentially guide therapeutic decisions.

The 10 articles in this Research Topic provide an overview of the current state of the art in ALS-FTD genetics and genomics, aiming to shed light on overlapping pathogenic mechanisms that may unite disparate mutations under a common umbrella and direct the search for disease-modifying therapies. We have learned much since the discovery of C9-linked ALS-FTD. The next decade promises to illuminate many new aspects of these overlapping neurodegenerative diseases. Building on multi-disciplinary efforts of international consortia such as Project MinE (www.projectmine.com), GENFI (http://genfi.org.uk/) and RiMOD-FTD (https://www.neurodegenerationresearch.eu/initiatives/annual-calls-for-proposals/closed-calls/risk-factors-2012/risk-factor-call-results/rimod-ftd/), we may begin to fully resolve ALS-FTD genetic architecture and understand why individuals carrying a particular variant go on to develop ALS, FTD, or ALS-FTD.

## Author Contributions

FC wrote the first draft. AR and HH critically reviewed the final version of this editorial. All authors approved the final version of this editorial.

## Conflict of Interest

The authors declare that the research was conducted in the absence of any commercial or financial relationships that could be construed as a potential conflict of interest.
